# Supplementary material for: Association of vaccine intention against COVID-19 using the 5C Scale and its constructs: a Pima County, Arizona cross-sectional survey
Source: PeerJ. 2024 Dec 6;12:e18316. doi: 10.7717/peerj.18316 (PMC11627084; doi:10.7717/peerj.18316)
Supplement: Supplemental Information 4 [file peerj-12-18316-s004.docx]

STROBE Statement—checklist of items that should be included in reports of observational studies

|  | Item No. | Recommendation | Page  No. | Relevant text from manuscript |
| --- | --- | --- | --- | --- |
| **Title and abstract** | 1 | (*a*) Indicate the study’s design with a commonly used term in the title or the abstract | 1-2 | **Title: Prediction of intention to vaccinate against COVID-19 using the 5C Scale and its constructs: A Pima County, Arizona cross-sectional survey**  **Methods.** From January to May 2021, we administered a cross-sectional survey…. |
|  |  | (*b*) Provide in the abstract an informative and balanced summary of what was done and what was found | 2 | **Methods.** From January to May 2021, we administered a cross-sectional survey among adults in Pima County, Arizona in collaboration with the local health department to assess psychological antecedents to COVID-19 vaccination using the 5C Scale. Participants were recruited virtually for the survey using multiple recruitment methods. Unadjusted and adjusted hierarchical ordinal logistic regressions were conducted to determine if the 5C variables had an association with intention to vaccinate against COVID-19.  **Results.** Of the 1,823 participants who responded to the survey, 924 (76%) were included in the final analyses. Respondents were White (71%), non-Hispanic (59%), Female (68%), Liberal (37%) and Married (46%). The average age of the participants was 43.9 (±1.3) years. Based on the 5C Scale, Confidence (adjOR:3.64, CI:3.08, 4.29), Collective Responsibility (adjOR:1.94, CI:1.57,2.39) and Complacency (adjOR:0.64, CI:0.51,0.80) were significantly associated with intention to vaccinate against COVID-19. |
| Introduction | | | |  |
| Background/rationale | 2 | Explain the scientific background and rationale for the investigation being reported | 3 | Vaccine hesitancy, often defined as the “delay in acceptance or refusal of vaccines despite availability of vaccine…. |
| Objectives | 3 | State specific objectives, including any prespecified hypotheses | 3 | we assessed if the 5C variables were associated with the outcome of intention to vaccinate against COVID-19 among adult Pima County residents.. |
| Methods | | | |  |
| Study design | 4 | Present key elements of study design early in the paper | 3-4 | Between January and May 2021, we implemented a cross-sectional sociobehavioral survey including the 5C Scale 5-item questionnaire among adults in Pima County, Arizona… |
| Setting | 5 | Describe the setting, locations, and relevant dates, including periods of recruitment, exposure, follow-up, and data collection | 3-4 | Between January and May 2021, we implemented a cross-sectional sociobehavioral survey including the 5C Scale 5-item questionnaire among adults in Pima County, Arizona.. |
| Participants | 6 | (*a*) *Cohort study*—Give the eligibility criteria, and the sources and methods of selection of participants. Describe methods of follow-up  *Case-control study*—Give the eligibility criteria, and the sources and methods of case ascertainment and control selection. Give the rationale for the choice of cases and controls  *Cross-sectional study*—Give the eligibility criteria, and the sources and methods of selection of participants | 4 | To participate in the study, potential participants had to be over the age of 18 years, have the ability to speak and read English or Spanish, report being a resident of Pima County, and have the ability to undergo informed consent process. |
|  |  | (*b*) *Cohort study*—For matched studies, give matching criteria and number of exposed and unexposed  *Case-control study*—For matched studies, give matching criteria and the number of controls per case |  |  |
| Variables | 7 | Clearly define all outcomes, exposures, predictors, potential confounders, and effect modifiers. Give diagnostic criteria, if applicable | 4-5 | The exposures of interest are the five domains listed in the five-item version of the 5C scale,…. |
| Data sources/ measurement | 8* | For each variable of interest, give sources of data and details of methods of assessment (measurement). Describe comparability of assessment methods if there is more than one group | 4-5 | Each item used a 5-point Likert scale scored Strongly Agree (1) to Strongly Disagree (5)…. |
| Bias | 9 | Describe any efforts to address potential sources of bias | 4 | Participants were recruited using several different strategies including the Qualtrics platform using stratified sampling methods with the intention of oversampling minorities. |
| Study size | 10 | Explain how the study size was arrived at | 4 | *A priori*, we aimed to recruit 1,000 participants to ensure we would have a size sufficient… |

Continued on next page

| Quantitative variables | 11 | Explain how quantitative variables were handled in the analyses. If applicable, describe which groupings were chosen and why | 4-5 | The exposures of interest are the five domains listed in the five-item version of the 5C scale, described by Betsch *et al*. as the psychological antecedents to vaccination (2020). The 5C items…. |
| --- | --- | --- | --- | --- |
| Statistical methods | 12 | (*a*) Describe all statistical methods, including those used to control for confounding | 5-6 | ***Statistical Analysis***  For descriptive statistics, univariate associations were examined using chi-square tests for all categorical demographic variables with the outcome variable of intention to vaccinate against… |
|  |  | (*b*) Describe any methods used to examine subgroups and interactions |  | NA |
|  |  | (*c*) Explain how missing data were addressed | 5 | Two of our researchers assessed each observation to determine if they had enough integrity to be kept in the final dataset used for analysis based on predetermined criteria including quantity of missing data… |
|  |  | (*d*) *Cohort study*—If applicable, explain how loss to follow-up was addressed  *Case-control study*—If applicable, explain how matching of cases and controls was addressed  *Cross-sectional study*—If applicable, describe analytical methods taking account of sampling strategy | 5-6 | Age, gender, race, ethnicity, self-identified political attitude, income, marital status, and education status have been shown to be associated with intention to vaccinate against seasonal influenza and COVID-19… |
|  |  | (*e*) Describe any sensitivity analyses | 6 | A sensitivity analysis was conducted by including all eligible data into the same analyses described… |
| Results | | | | |
| Participants | 13* | (a) Report numbers of individuals at each stage of study—eg numbers potentially eligible, examined for eligibility, confirmed eligible, included in the study, completing follow-up, and analysed | 6 | Although 1,823 participants started the questionnaire, only 1,219 (67% of total) participants were eligible to complete the survey, 1,194 participants successfully completed the survey… |
|  |  | (b) Give reasons for non-participation at each stage | 6 | Possible reasons for nonparticipation could include survey fatigue or the use of bots to complete data automatically. |
|  |  | (c) Consider use of a flow diagram | 6 | Figure 1 |
| Descriptive data | 14* | (a) Give characteristics of study participants (eg demographic, clinical, social) and information on exposures and potential confounders | 6-7 | *Description of the data*  The largest representation of our participants was female (68%), non-Hispanic (59%), married…  Table 2 |
|  |  | (b) Indicate number of participants with missing data for each variable of interest |  | Not possible since much of the missing data did not complete a significant portion of the survey |
|  |  | (c) *Cohort study*—Summarise follow-up time (eg, average and total amount) |  |  |
| Outcome data | 15* | *Cohort study*—Report numbers of outcome events or summary measures over time |  |  |
|  |  | *Case-control study—*Report numbers in each exposure category, or summary measures of exposure |  |  |
|  |  | *Cross-sectional study—*Report numbers of outcome events or summary measures | 6-7 | *Description of the data*  The largest representation of our participants was female (68%), non-Hispanic (59%), married…  Table 2 |
| Main results | 16 | (*a*) Give unadjusted estimates and, if applicable, confounder-adjusted estimates and their precision (eg, 95% confidence interval). Make clear which confounders were adjusted for and why they were included | 7 | In the hierarchical regression, characteristics that were immutable, such as demographic characteristics, were entered first. Then, based on binary correlations, the significant… |
|  |  | (*b*) Report category boundaries when continuous variables were categorized | 7 | In the hierarchical regression, characteristics that were immutable, such as demographic characteristics, were entered first. Then, based on binary correlations, the significant… |
|  |  | (*c*) If relevant, consider translating estimates of relative risk into absolute risk for a meaningful time period |  | NA |

Continued on next page

| Other analyses | 17 | Report other analyses done—eg analyses of subgroups and interactions, and sensitivity analyses | 7 | In the sensitivity analysis, when all eligible and previously excluded observations were included (n=1,194), |
| --- | --- | --- | --- | --- |
| Discussion | | | | |
| Key results | 18 | Summarise key results with reference to study objectives | 7-8 | In our study, Confidence and Collective Responsibility were significantly positively associated with self-reported intention to vaccinate in the future against COVID-19, while Complacency… |
| Limitations | 19 | Discuss limitations of the study, taking into account sources of potential bias or imprecision. Discuss both direction and magnitude of any potential bias | 8 | A limitation of the study is that the sampling methods relied upon convenience and snowball sampling, leading to some possible bias in the sample. Additionally, no survey weights were used in the analysis to account for oversampling of minority populations, making the findings… |
| Interpretation | 20 | Give a cautious overall interpretation of results considering objectives, limitations, multiplicity of analyses, results from similar studies, and other relevant evidence | 8 | Findings from this study can be used to help inform vaccination promotion efforts, considering…  In conclusion, in this study we hypothesized that Confidence and Collective Responsibility would be the most highly correlated variables with the outcome of intention to… |
| Generalisability | 21 | Discuss the generalisability (external validity) of the study results | 8 | The sample is also limited to Pima County which may limit the findings’ generalizability… |
| Other information | |  | | |
| Funding | 22 | Give the source of funding and the role of the funders for the present study and, if applicable, for the original study on which the present article is based | 9 | **Funding**  This study was conducted using PM’s start-up funds… |

*Give information separately for cases and controls in case-control studies and, if applicable, for exposed and unexposed groups in cohort and cross-sectional studies.

**Note:** An Explanation and Elaboration article discusses each checklist item and gives methodological background and published examples of transparent reporting. The STROBE checklist is best used in conjunction with this article (freely available on the Web sites of PLoS Medicine at http://www.plosmedicine.org/, Annals of Internal Medicine at http://www.annals.org/, and Epidemiology at http://www.epidem.com/). Information on the STROBE Initiative is available at www.strobe-statement.org.
