# Supplementary material for: Association of vaccine intention against COVID-19 using the 5C Scale and its constructs: a Pima County, Arizona cross-sectional survey
Source: PeerJ. 2024 Dec 6;12:e18316. doi: 10.7717/peerj.18316 (PMC11627084; doi:10.7717/peerj.18316)
Supplement: Supplemental Information 5 [file peerj-12-18316-s005.docx]

**Appendix A:** Questions from questionnaire in 5C Scale Study

| **Item** | **Options** | **Type** |
| --- | --- | --- |
| **Confidence** I am completely confident that COVID-19 vaccines are safe. | 1, Strongly Disagree \| 2, Disagree \| 3, Neither Agree nor Disagree \| 4, Agree \| 5, Strongly Agree | Categorical |
| **Complacency** Vaccination is not necessary because COVID-19 is not common anymore. | 1, Strongly Disagree \| 2, Disagree \| 3, Neither Agree nor Disagree \| 4, Agree \| 5, Strongly Agree | Categorical |
| **Constraints** Everyday stress prevents me from getting vaccinated against COVID-19. | 1, Strongly Disagree \| 2, Disagree \| 3, Neither Agree nor Disagree \| 4, Agree \| 5, Strongly Agree | Categorical |
| **Calculation** When I think about getting vaccinated for COVID-19, I weigh benefits and risks to make the best decision possible. | 1, Strongly Disagree \| 2, Disagree \| 3, Neither Agree nor Disagree \| 4, Agree \| 5, Strongly Agree | Categorical |
| **Collective responsibility** When everyone is vaccinated for COVID-19, I don’t have to get vaccinated, too. | 1, Strongly Agree \| 2, Agree \| 3, Neither Agree nor Disagree \| 4, Disagree \| 5, Strongly Disagree | Categorical |
| **Gender** | 1, Male \| 2, Female \| 3, Nonbinary/third gender, Prefer not to say, Other | Categorical |
| **Age** | Continuous variable, | Categorical |
| **Ethnicity** Are you Hispanic or Latino? | 1, Yes \| 2, No \| 99, Don't Know/Not Sure, Prefer not to say | Categorical |
| **Race** What is your race? Do you consider yourself... (Select one or more.) | 1, White \| 2, Black / African American \| 3, Asian \| 4, Native Hawaiian, other Pacific Islander, American Indian or Alaska Native \| 5, Other \|7, Mixed race, \| 99, Prefer not to say | Categorical |
| **Marital Status** | 0, Single \| 1, Married, Member of unmarried couple \| 2, Divorced, Widowed, Separated, Never married \| 99, Prefer not to say | Categorical |
| **Education level** What is the highest grade or year of school you have completed? | 0, below college graduate \| 1, College Graduate \| 99, prefer not to say | Categorical |
| **Income** Please select your income range | 1, Less than $25,000 \| 2, $25,000 to less than $50,000 \| 3, $50,000 to less than $75,000 \| 4, $75,000 or more \| 99, Prefer not to say | Categorical |
| **Political status** What is your political affiliation? | 1, Liberal \| 2, Moderate \| 3, Prefer not to say \| 4, Conservative | Categorical |
| **Intention** How likely is it that you will get a COVID-19 vaccine (again) in the future? | 1, Extremely unlikely \| 2, Unlikely \| 3, Neither likely nor unlikely \| 4, Likely \| 5, Extremely likely | Categorical |
