# Supplementary material for: Association of vaccine intention against COVID-19 using the 5C Scale and its constructs: a Pima County, Arizona cross-sectional survey
Source: PeerJ. 2024 Dec 6;12:e18316. doi: 10.7717/peerj.18316 (PMC11627084; doi:10.7717/peerj.18316)
Supplement: Supplemental Information 6 [file peerj-12-18316-s006.docx]

**Appendix B.** Sensitivity analysis including all eligible observations – a hierarchical ordinal logistic regression models for 5C scale’s correlation to Pima County adults’ intention to vaccinate (n= 1,194)

|  | **Unadjusted**  OR (95% CI) | **Model 0/Dem**  OR (95% CI) | **Model 1/Conf**  OR (95% CI) | **Model 2/ Adjusted***  OR (95% CI) |  |
| --- | --- | --- | --- | --- | --- |
| *Sensitivity analysis, n=1,194* | | | | |  |
| **Age*** | -- | **1.19 (1.10, 1.30)** | **1.16 (1.06,1.28)** | 1.05 (0.96,1.16) |  |
| **Gender (Male)**  Female  Nonbinary/Other/PNTS** | -- | **0.69 (0.54, 0.87)**  1.62 (0.87, 3.03) | **0.51 (0.40, 0.66)**  0.97 (0.48, 1.96) | **0.69 (0.52, 0.90)**  2.02 (0.95, 4.29) |  |
| **Ethnicity (Non-Hisp)**  Hispanic  PNTS** | -- | 0.90 (0.70, 1.17)  0.90 (0.39, 2.04) | 0.76 (0.57, 1.00)  0.54 (0.22, 1.32) | 0.90 (0.67, 1.20)  0.66 (0.26, 1.63) |  |
| **Race (White)**  Black/AA**  Asian  Indigenous**  Other Race  Mixed  PNTS** | -- | **0.54 (0.33, 0.87)**  1.14 (0.70, 1.84)  **0.57 (0.40, 0.80)**  0.85 (0.54, 1.35)  **0.30 (0.18, 0.50)**  1.61 (0.70, 3.71) | **0.59 (0.36, 0.98)**  1.01 (0.59, 1.71)  **0.64 (0.44, 0.93)**  0.98 (0.60, 1.61)  **0.54 (0.31, 0.94)**  1.63 (0.69, 3.85) | 0.78 (0.45, 1.34)  1.10 (0.64, 1.90)  0.84 (0.57, 1.24)  0.97 (0.58, 1.63)  **0.52 (0.30, 0.91)**  1.51 (0.63, 3.63) |  |
| **Education (Not Grad)**  College graduate  PNTS** | -- | 1.47 (1.13, 1.91)  0.77 (0.24, 2.45) | 1.26 (0.95, 1.66)  0.56 (0.17, 1.81) | 1.11 (0.83, 1.49)  1.48 (0.44, 4.92) |  |
| **Marital Status (Single)**  Married/Partnered  Div/Sep/Wid**  PNTS** | -- | **0.70 (0.53, 0.93)**  **0.57 (0.38, 0.84)**  **0.27 (0.09, 0.80)** | 0.78 (0.57, 1.06)  **0.61 (0.40, 0.93)**  0.49 (0.16, 1.49) | 1.09 (0.80, 1.50)  0.91 (0.59, 1.40)  0.60 (0.20, 1.81) |  |
| **Income (<25K USD/yr)**  25,000–49,999  50 – 74,999  >75,000  PNTS** | -- | 1.22 (0.91, 1.63)  1.22 (0.83, 1.78)  **1.90 (1.24, 2.92)**  1.44 (0.87, 2.39) | 0.83 (0.60, 1.14)  1.03 (0.69, 1.55)  1.52 (0.96, 2.41)  0.93 (0.54, 1.59) | 0.86 (0.62, 1.20)  0.84 (0.55, 1.27)  1.31 (0.81, 2.12)  0.94 (0.53, 1.67) |  |
| **Political aff. (Liberal)**  Moderate  Conservative  PNTS** | -- | **0.53 (0.40, 0.69)**  **0.30 (0.21, 0.43)**  **0.26 (0.19, 0.37)** | **0.59 (0.44, 0.81)**  **0.44 (0.30, 0.65)**  **0.41 (0.28, 0.59)** | 0.79 (0.58, 1.09)  **0.45 (0.30, 0.67)**  **0.62 (0.42, 0.91)** |  |
|  | | | | |  |
| **Confidence** | **3.79 (3.29, 4.36)** | -- | **4.10 (3.57, 4.69)** | **3.73 (3.23, 4.32)** |  |
| **Coll responsibility** | **1.99 (1.67, 2.38)** | -- | -- | **2.02 (1.68, 2.42)** |  |
| **Complacency** | **0.60 (0.50, 0.72)** | -- | -- | **0.63 (0.52, 0.76)** |  |
| **Constraints** | 1.00 (0.87, 1.15) | -- | -- | 1.06 (0.91, 1.24) |  |
| **Calc of risk** | 1.09 (0.96, 1.23) | -- | -- | 1.07 (0.94, 1.22) |  |
|  |  |  |  |  |  |
| **Pseudo R^2^** | 0.29 | 0.07 | 0.24 | 0.30 |  |

*Note: bolded text means that the p-value was less than 0.05. Abbreviations: OR=odds ratios; 95% CI = 95% Confidence intervals. Reference groups for categorical categories are single for marital status, less than college graduate for education, and liberal for political status.*

** Age ORs are for every 10 years.*

*** Indigenous= Native Hawaiian or other Pacific Islander or American Indian or Alaska Native, AA= African American, Div/Sep/Wid = Divorced, Separated, Widowed, PNTS= Prefer not to say*
